# Autoantibodies in Wilson disease: Impact on clinical course

**DOI:** 10.1002/jmd2.12317

**Published:** 2022-07-22

**Authors:** Magdalena Antczak‐Kowalska, Anna Członkowska, Ceren Eyileten, Anna Palejko, Agnieszka Cudna, Marta Wolska, Agnieszka Piechal, Tomasz Litwin

**Affiliations:** ^1^ 2nd Department of Neurology Institute of Psychiatry and Neurology Warsaw Poland; ^2^ Center for Preclinical Research and Technology CEPT, Department of Experimental and Clinical Pharmacology Medical University of Warsaw Warsaw Poland; ^3^ Doctoral School, Medical University of Warsaw Warsaw Poland

**Keywords:** antineuronal antibodies, anti‐neutrophil cytoplasmic antibodies, antinuclear antibodies, autoantibodies, drug‐induced antibodies, Wilson disease

## Abstract

Symptoms of Wilson disease (WD) vary and additional factors such as autoimmunity may play an important role in WD pathogenesis. The presence of antinuclear antibodies (ANA), anti‐neutrophil cytoplasmic antibodies, neuronal surface antibodies, and onconeural antibodies in WD was investigated using standardized indirect immunofluorescence assays and Western Blot analysis. The presence of all studied autoantibodies was higher in WD patients in comparison to healthy subjects, but there was no statistically significant difference in autoantibodies frequency according to disease manifestation. D‐penicillamine treatment was associated with a higher presence of ANA than zinc sulfate but without an increase in autoimmune diseases rate.

**SYNOPSIS**

Elevated presence of autoantibodies in WD is not related to the clinical manifestations of WD and may represent a bystander phenomenon, though therapy with D‐penicillamine increased ANA presence, but not overt autoimmune diseases.

## 1 INTRODUCTION

Wilson disease (WD) is a rare, autosomal recessive disorder of copper metabolism, with an estimated prevalence of 1:30 000–1:50 000, which is higher in some isolated communities (e.g., 1:8700 in Sardinia, 1:19 000 in Costa Rica).^1^ WD is caused by mutations in the gene encoding the copper‐transporting enzyme ATP7B, leading to copper overload in the liver, brain and other organs.^2^ The clinical symptoms of WD can generally be divided into hepatic or neurological/psychiatric manifestations. The hepatic signs are not specific, but include progressive hepatic failure and cirrhosis with ascites and esophageal varices. The neurological presentation, which may range from very subtle to rapid in progression, typically includes tremor, dysarthria, ataxia and dystonia. Psychiatric symptoms may range from behavioral change and depression to psychosis. Importantly, WD can be asymptomatic, especially in the early stages, and is often diagnosed during family screening or incidentally.^2^, ^3^, ^4^ The general goal of WD treatment is to establish normal copper homeostasis and prevent development or progression of symptoms.^5^ Commonly used medications include D‐penicillamine, which is a copper chelator that increases urinary copper excretion, and zinc salts, which decrease copper absorption in the gastrointestinal tract.^2^, ^3^

Synthesis of autoantibodies is a common feature of systemic autoimmune diseases and their variability and amount are important diagnostic and prognostic factors.^6^ Moreover, autoantibodies can occur in healthy individuals or in diseases caused by other mechanisms.^6^, ^7^ Also, it was already reported that multiple drugs can induce autoimmunity and clinically overt autoimmune syndromes in some cases.^8^, ^9^, ^10^ D‐penicillamine is one such drug that can induce autoantibody production, including antinuclear antibodies (ANA), anti‐neutrophil cytoplasmic antibodies (ANCA), anti‐muscle‐specific tyrosine kinase antibodies and anti‐acetylcholine receptor antibodies.^11^, ^12^, ^13^ Moreover, multiple liver diseases, including autoimmune hepatitis, influence the immune system and lead to increased serum autoantibody production.^14^ Consequently, due to liver injury, as well as D‐penicillamine treatment, we may expect elevated prevalence of autoantibodies in patients with WD. A previous study showed the presence of autoimmunization in WD patients, with frequent elevation of ANA levels that relatively rarely lead to clinically overt autoimmune diseases during treatment with D‐penicillamine.^15^ These results show some immunological background in WD and suggest the possibility of autoimmunization to other autoantigens that may influence the central nervous system (CNS) and modify the course of WD. Antineuronal antibodies have been recently associated with various CNS pathologies, including movement disorders, seizures, rapid cognitive decline and psychiatric symptoms.^16^

The aim of this study was to investigate the presence of autoantibodies (ANA, ANCA, neuronal surface antibodies [NSAbs] and onconeural antibodies [ONA]) in patients with WD and analyze the relationship between the tested autoantibodies and the clinical manifestation of WD. Moreover, we assessed the effect of administered treatment on autoantibody synthesis.

## 2 METHODOLOGY

### 2.1 Study population

In total, 88 patients with confirmed WD and 100 healthy individuals were included in the study. All participants were examined at the 2nd Department of Neurology and Outpatient Clinic, Institute of Psychiatry and Neurology, Warsaw, Poland. Patients were diagnosed with WD based on a laboratory confirmation of abnormal copper metabolism, presence of a Kayser‐Fleischer ring, genetic analysis for known mutations and, in doubtful cases, with ^64^Cu radioactive test performed in our center, as previously described.^17^ The Unified Wilson's Disease Rating Scale (UWDRS) was used for the neurological assessment, including Part II (disability, based on the Barthel Scale) and Part III (detailed neurological examination).^18^ According to the results of the clinical examination and additional tests (basic laboratory liver tests, ultrasound examination of the liver and brain magnetic resonance imaging), patients were classified into three groups based on the form of presentation: hepatic, neurological or asymptomatic.^19^ After diagnosis, patients were administered either D‐penicillamine or zinc sulfate in standard doses, the decision about which drug to select was made individually by attending neurologists and based on their clinical experience and patients' preferences.^20^ Patients enrolled in the study received treatment for at least 6 months. In a subgroup of 36 patients, there were also accessible clinical data and samples of serum taken at the time of diagnosis. The control group included 100 age‐ and sex‐matched healthy volunteers with no history of WD, liver disease, neurological or mental disease, chronic inflammatory disease or infectious disease.

The study was conducted in accordance with the current version of the Declaration of Helsinki and informed written consent was obtained from each participant. The Ethics Committee of the Institute of Psychiatry and Neurology approved both the study protocol and the informed consent form.

### 2.2 Sample collection and determination of antibodies

Blood samples were taken during routine, initial or follow‐up examinations. Patients were monitored, including biochemical testing, for the development of neurological or hepatic symptoms. Blood samples were taken in the morning and centrifuged at 1500*g* for 15 min at 18–25°C and stored at −70°C until examination. Serum specimens were screened by standardized immunofluorescence assays and Western Blot analysis for immunoglobulin G with the following specificities: ANA (nRNP/Sm, Sm, SSA, Ro‐52, SSB, Scl‐70, PM‐Scl, PCNA, Jo‐1, CENP‐B, dsDNA, nucleosomes, histones, ribosomal protein‐P, AMA‐M2, and DFS‐70) with indirect immunofluorescence assay and Western blot; ANCA (pANCA and cANCA) with indirect immunofluorescence assay; NSAb (NMDA, AMPA1, AMPA2, LGI1, CASPR2, and GABAB) with indirect immunofluorescence assay; and ONA (Hu, Yo, Ri, anti‐amphiphysin, CV2, Ma2/Ta, recoverin, SOX1, Zic4, titin, GAD65, and Tr) with Western blot. The presence of autoantibodies was considered positive if they were detected in titers ≥1:10 for ANCA and NSAb, ≥1:100 for ANA and detectable at qualitative assay (dilution 1:101) for ONA. Autoantibodies were detected using commercial kits according to manufacturers' protocols (Euroimmun, Lubeck, Germany).

### 2.3 Statistical analysis

All results for categorical variables were presented as number and percentage. Continuous variables were expressed as mean and standard deviation (SD) or median and interquartile range (IQR), depending on the normality of the distribution, assessed with the use of the Shapiro–Wilk test. Continuous variables were compared using the Student's *t*‐test or the Mann–Whitney test, according to their distribution. Categorical data were analyzed with the Chi‐square test. Paired samples comparisons were calculated with the McNemar test. All tests were two‐sided with the significance level of *p* < 0.05. Calculations were performed using Statistica 13.1 software (Dell Inc., Tulsa, OK).

## 3 RESULTS

### 3.1 Patients demographics

The study group included 88 WD patients, 40 men (45.5%) and 48 women (54.5%), with a mean age of 38.4 ± 10.7 years, which was not significantly different from control subjects (Table 1). The H1069Q mutation was detected in 84.09% (*n* = 74) of patients, 48.86% of patients (*n* = 43) were homozygous for this mutation, 21.59% of patients (*n* = 19) were compound heterozygotes, and in 13.64% of cases (*n* = 12) mutation was detected on only one allele; 14 patients (15.91%) had non‐H1069Q mutations. Of the analyzed patients, 66% (*n* = 58) presented with neurological manifestations and 27% (*n* = 24) had hepatic manifestations, whereas 7% (*n* = 6) were asymptomatic. The mean ± SD UWDRS scores for patients with neurological manifestation were UWDRS II 9.05 ± 1.78 and UWDRS III 31.09 ± 4.28. In total, 53% (*n* = 47) were treated with D‐penicillamine and 47% (*n* = 41) with zinc sulfate. The median time of treatment was 2.25 years (IQR = 8.17). As expected in patients with WD, laboratory tests showed abnormalities demonstrating liver pathology. Aspartate transaminase, alanine transaminase, gamma‐glutamyl transpeptidase, bilirubin and International Normalized Ratio were significantly elevated in comparison to healthy individuals.

**TABLE 1:** Participants characteristics

	Patients with WD	Healthy individuals	
	*n* = 88	*n* = 100	*p*
Age (years)	38.4 ± 10.7	37.6 ± 9.8	0.605
Sex (male)	40 (45.5%)	49 (49.0%)	0.627
Treatment			
D‐penicillamine	47 (53%)	—	—
Zinc sulfate	41 (47%)	—	—
Duration of treatment (years)	2.25 (8.04)	—	—
Laboratory data			
White blood cell count (×10^9^/L)	5.25 ± 2.3	6.38 ± 1.90	**0.001**
Platelets (×10^9^/L)	160.56 ± 69.40	225.03 ± 39.27	**<0.001**
AST (IU/L)	32.92 ± 36.81	22.48 ± 9.54	**0.007**
ALT (IU/L)	25.70 (19.25)	16.20 (12.55)	**0.009**
GGTP (IU/L)	27.70 (40.53)	16.40 (13.3)	**0.002**
Bilirubin (mg/dL)	0.77 (0.53)	0.59 (0.29)	**<0.001**
INR	1.18 ± 0.15	1.07 ± 0.09	**<0.001**

Abbreviations: ALT, alanine transaminase; AST, aspartate transaminase; GGTP, gamma‐glutamyl transpeptidase; INR, International Normalized Ratio; UWDRS, Unified Wilson's Disease Rating Scale; WD, Wilson disease.

*Note*: Data are presented as number (percentage) or mean ± standard deviation or median (interquartile range) depending on the distribution of the data. *p* values marked with bold indicate statistically significant differences between the groups <0.05.

In the subgroup of 36 treatment‐naïve patients, 47.2% of patients were administered D‐penicillamine and 52.8% zinc sulfate and median time of treatment was 2.0 years (IQR = 0.08). Follow‐up laboratory examinations showed significant improvement of liver function tests after treatment (data not shown).

Concomitant autoimmune diseases were noted in the medical records of 4 patients (hypothyroidism, *n* = 3; rheumatoid arthritis, *n* = 1); 2 were treated with D‐penicillamine and 2 with zinc sulfate; 3 had hepatic manifestations and 1 neurological manifestations. In one case, autoimmune disease was known before WD was diagnosed, and in the other three cases, conditions were diagnosed while patients were on treatment.

### 3.2 Autoantibody presence

The presence of all studied autoantibodies was higher in patients with WD in comparison to healthy subjects (ANA 21.6% vs 11.0%, *p* = 0.048; ANCA 28.4% vs 15.0%, *p* = 0.025; NSAbs 9.1% vs 2.0%, *p* = 0.031; ONA 13.6% vs 5.0%, *p* = 0.039) (Table 2). Patients carrying the H1069Q mutation had higher frequency of autoantibodies as compared with non‐H1069Q patients, but these differences reached statistical significance only in the case of ANA (25.68% vs 0.00%, *p* = 0.032) (Table 3). There were no statistically significant differences in the frequency of autoantibodies between H1069Q homozygotes, compound heterozygotes and one allele mutation carriers (data not shown). Female sex was associated with significantly higher presence of ANA in comparison to males, both in WD (31.3% vs 10.0%, *p* = 0.016) and in healthy individuals (17.6% vs 4.1%, *p* = 0.030); for other analyzed antibodies, sex differences were not observed (data not shown). Detailed distribution of detected autoantibodies is presented in Table 4, with no statistical analyses due to the small numbers of patients.

**TABLE 2:** Autoantibody presence in healthy controls and patients with WD

				WD manifestation				Treatment		
	Controls (*n* = 100)	All WD patients (*n* = 88)	*p*	Hepatic (*n* = 24)	Neurological (*n* = 58)	Asymptomatic (*n* = 6)	*p*	D‐penicillamine (*n* = 47)	Zinc sulfate (*n* = 41)	*p*
Treatment duration (years)	—	2.25 (8.04)	—	2.08 (5.48)	2.88 (8.63)	2.00 (0.00)	0.739	2.58 (3.50)	2.08 (9.92)	0.664
ANA, *n* (%)	11 (11.0%)	19 (21.6%)	**0.048**	2 (8.3%)	14 (24.1%)	3 (50.0%)	0.062	14 (29.8%)	5 (12.2%)	**0.045**
ANCA, *n* (%)	15 (15.0%)	25 (28.4%)	**0.025**	5 (20.8%)	19 (32.8%)	1 (16.7%)	0.444	12 (25.5%)	13 (31.7%)	0.522
NSAbs, *n* (%)	2 (2.0%)	8 (9.1%)	**0.031**	2 (8.3%)	4 (6.9%)	2 (33.3%)	0.099	4 (8.5%)	4 (9.8%)	0.839
ONA, *n* (%)	5 (5.0%)	12 (13.6%)	**0.039**	2 (8.3%)	9 (15.5%)	1 (16.7%)	0.672	3 (6.4%)	9 (21.9%)	**0.034**

Abbreviations: ANA, antinuclear antibodies; ANCA, anti‐neutrophil cytoplasmic antibodies; NSAb, neuronal surface antibodies; ONA, onconeural antibodies; WD, Wilson disease.

*Note*: Data are presented as number (percentage) or median (interquartile range). *p* values marked with bold indicate statistically significant differences between the groups <0.05.

**TABLE 3:** Autoantibody presence in patients with H1069Q and non‐H1069Q mutations

	H1069Q mutation *n* = 74 (84.09%)	Non‐H1069Q mutations *n* = 14 (15.91%)	
	Homozygotes *n* = 43 (48.86%)	Homozygotes *n* = 4 (4.55%)	
	Compound heterozygotes *n* = 19 (21.59%)	Compound heterozygotes *n* = 3 (3.41%)	
	Mutation on one allele *n* = 12 (13.64%)	Mutation on one allele *n* = 3 (3.41%)	
		Unknown mutation *n* = 4 (4.55%)	*p*
ANA, *n* (%)	19 (25.68%)	0 (0.00%)	**0.032**
ANCA, *n* (%)	24 (32.43%)	1 (7.14%)	0.054
NSAbs, *n* (%)	7 (9.46%)	1 (7.14%)	0.782
ONA, *n* (%)	11 (14.86%)	1 (7.14%)	0.440

Abbreviations: ANA, antinuclear antibodies; ANCA, anti‐neutrophil cytoplasmic antibodies; NSAb, neuronal surface antibodies; ONA, onconeural antibodies; WD, Wilson disease.

*Note*: Data are presented as number (percentage). *p* values marked with bold indicate statistically significant differences between the groups <0.05.

**TABLE 4:** Detailed autoantibody distribution

	WD patients		Healthy individuals	
	Antibody	*n*	Antibody	*n*
ANA	nRNP/Sm	1	PCNA	2
	Ro‐52	1	Jo‐1	1
	Scl‐70	2	AMA‐M2	1
	PCNA	3	DFS‐70	8
	CENP‐B	1		
	dsDNA	8		
	Histones	1		
	Ribosomal protein‐P	2		
	AMA‐M2	2		
	DFS‐70	4		
ANCA	p‐ANCA	18	p‐ANCA	8
	c‐ANCA	7	c‐ANCA	7
NSAbs	NMDA	7	NMDA	1
	LGI1	1	CASPR2	1
ONA	Ma2/Ta	1	Hu	1
	Zic4	1	Yo	2
	Recoverin	1	Recoverin	1
	GAD65	2	Titin	1
	Yo	4		
	Titin	2		
	Anti‐amphiphysin	1		

Abbreviations: ANA, antinuclear antibodies; ANCA, anti‐neutrophil cytoplasmic antibodies; NSAb, neuronal surface antibodies; ONA, onconeural antibodies; WD, Wilson disease.

*Note*: Data are presented as number.

### 3.3 Relationship between autoantibodies and WD disease manifestations

Differences in the presence of autoantibodies in relation to WD manifestations did not reach statistical significance. However, in patients presenting neurological manifestations, the presence of autoantibodies was significantly elevated in comparison to healthy individuals: ANA (24.1% vs 11.0%, *p* = 0.029), ANCA (32.8% vs 15.0%, *p* = 0.009), and ONA (15.5% vs 5.0%, *p* = 0.025), with no significant differences observed for patients with hepatic manifestations (Table 2).

In the group of patients receiving the treatment, UWDRS II and III scores were evaluated during follow‐up and the presence of ANCA was associated with slightly higher UWDRS II score in comparison to patients without ANCA (*p* = 0.050). There was no significant relationship between the presence of ANA, NSAbs and ONA and UWDRS scores (data not shown). No significant correlations were found between the presence of autoantibodies and baseline evaluation of the UWDRS II and III scores in treatment‐naive patients (data not shown).

### 3.4 Relationship between autoantibodies and administered treatment

The presence of autoantibodies in relation to the administered treatment is presented in Table 2. In patients treated with D‐penicillamine, ANA were observed more frequently than in healthy individuals (29.8% vs 11.0%, *p* = 0.005), whereas in zinc sulfate‐treated patients, ANCA, NSAbs, and ONA were significantly more prevalent than in healthy individuals (31.7% vs 15.0%, *p* = 0.024, 9.8% vs 2.0%, *p* = 0.038, 21.9% vs 5.0%, *p* = 0.002, respectively). There was also statistically significant differences between D‐penicillamine‐ and zinc sulfate‐treated groups for the presence of ANA (29.8% vs 12.2%, *p* = 0.045) and ONA (6.4% vs 21.9%, *p* = 0.034).

In the subgroup of 36 treatment‐naïve patients, the presence of autoantibodies did not increase significantly after 2 years median follow‐up and there was also no difference in autoantibodies presence in relation to administered treatment (Table 5).

**TABLE 5:** Presence of autoantibodies in newly diagnosed patients, before and after a median duration of treatment of 2 years

	All patients			D‐penicillamine			Zinc sulfate		
	*n* = 36			*n* = 17			*n* = 19		
	At diagnosis	During treatment	*p*	At diagnosis	During treatment	*p*	At diagnosis	During treatment	*p*
ANA	3 (8.3%)	6 (16.7%)	0.450	1 (5.9%)	3 (17.7%)	0.617	2 (10.5%)	3 (15.8%)	1.000
ANCA	7 (19.4%)	7 (19.4%)	0.752	2 (11.8%)	1 (5.9%)	1.000	5 (26.3%)	6 (31.6%)	1.000
NSAbs	1 (2.8%)	3 (8.3%)	0.480	1 (5.9%)	1 (5.9%)	—	0 (0.0%)	2 (10.5%)	—
ONA	2 (5.6%)	3 (8.3%)	1.000	0 (0.0%)	0 (0.0%)	—	2 (10.5%)	3 (15.8%)	1.000

Abbreviations: ANA, antinuclear antibodies; ANCA, anti‐neutrophil cytoplasmic antibodies; NSAbs, neuronal surface antibodies; ONA, onconeural antibodies.

*Note*: Data are presented as number (percentage).

## 4 DISCUSSION

In our study, the presence of all studied autoantibodies was higher in WD patients in comparison to healthy subjects, but there was no statistically significant difference in antibodies frequency between subgroups presenting neurological and hepatic manifestations of WD.

Some immunological abnormalities were demonstrated in untreated WD patients, increased levels of IgG and IgM, as well as decreased T cell‐mediated immunity were observed, which were similar to those observed in patients with liver cirrhosis of other causes.^21^ After treatment with D‐penicillamine, the immunological response predominantly normalized, which may be due to improved liver function and decreased levels of toxic free copper.^22^ In addition, serum interleukin‐6, tumor necrosis factor (TNF)‐α and interferon‐γ levels were elevated in WD patients, which may indicate ongoing inflammatory process.^23^ It is believed that neuroinflammation may play an important role in the pathogenesis of WD.^24^, ^25^ Although CNS has often been considered as immune‐privileged and isolated from the peripheral immune system by the blood–brain barrier, immune mediators released in the periphery can pass through the blood–brain barrier,^26^ showing direct neurotoxicity and leading to microglia and astrocyte activation.^27^ Also relevant to WD, advanced cirrhosis, regardless of its etiology, may lead to cirrhosis‐associated immune dysfunction, immunodeficiency and systemic inflammation, activation of circulating immune cells and increase in serum levels of pro‐inflammatory cytokines.^28^

D‐penicillamine is associated with increased risk of autoimmune diseases, with drug‐induced lupus, vasculitis, myasthenia gravis, glomerulonephritis, epidermolysis bullosa acquisita noted in many case reports.^12^, ^29^, ^30^, ^31^, ^32^, ^33^ In 235 WD patients, coexisting autoimmune disorders were found in 8.1% of the patients, with 5.5% having pre‐existing autoimmune diseases and 2.6% developing an autoimmune condition while receiving long‐term D‐penicillamine treatment.^15^ In our study, clinically overt autoimmune diseases were rare (4 cases) and appeared unrelated to treatment type.

Detection of ANA is important in the diagnosis of autoimmune diseases, such as connective tissue diseases^34^ and autoimmune hepatitis.^35^, ^36^ Furthermore, ANA can be detected in almost 25% of the general population, more commonly in women and the elderly.^34^ In our analysis, there was significantly higher prevalence of ANA in females than in males both in WD patients and control subjects. The knowledge about the significance of immunological reactions and ANA in WD is limited. The role of ANA in therapy monitoring in 235 patients with WD was evaluated in a retrospective study by Seessle et al.^15^ Elevations in ANA were observed frequently, but there was no correlation between ANA titer and the development of concomitant autoimmune diseases, as well as WD manifestations. The study showed that the higher prevalence of ANA in WD patients was rather dependent on long‐term treatment than other causes.^15^ ANA were detected in 19% of patients in the study by Seessle et al, which is a similar prevalence to that seen in the general Polish population.^37^ In our analysis, we found that ANA were significantly more prevalent in WD patients compared to healthy subjects, suggesting the presence of some autoimmune alterations in WD. Furthermore, D‐penicillamine treatment was associated with elevation in the frequency of ANA compared with zinc sulfate, but with no increase in the rate of clinically overt autoimmune diseases.

Various drugs including D‐penicillamine can trigger ANCA synthesis and induce vasculitis.^38^ Detection of ANCA in the autoimmune diseases, particularly, connective tissue diseases, is frequently related with more clinical complications and treatment ineffectiveness, even if there are no signs of vasculitis and thus it can be presumed that ANCA present the final expression of an immune dysregulation, rather than a causative agent for the observed organs injury.^39^

Importantly, only a few studies have shown the impact of pathogenic ANCA in WD. Some case reports documented that D‐penicillamine treatment induced ANCA‐associated vasculitis and glomerulonephritis in patients with WD.^12^, ^30^, ^32^ Our analysis showed elevated prevalence of ANCA in WD patients in comparison to healthy individuals, but with no association with disease manifestation. Vasculitis is not a part of pathomorphological features of WD known from postmortem brain examinations.^40^ The significance of increased ANCA in WD is unclear, but may possibly reflect immunological dysregulation and a bystander phenomenon.

There are reports suggesting relatively frequent presence of NSAbs in patients with slowly progressing cognitive impairment or movement disorders mimicking neurodegenerative diseases, for example, Alzheimer disease (LGI1),^41^, ^42^ frontotemporal dementia (voltage‐gated potassium channels, VGKC‐Abs),^43^ amyotrophic lateral sclerosis (GABAB),^44^ and several NSAbs were found in patients diagnosed with Creutzfeldt–Jakob disease.^45^ In a recent study, NSAbs (GlyR, GABAAR, LGI1, CASPR2, and GABABR) were detected in 13.8% (*n* = 13) of patients with dementia and parkinsonism, mostly presenting with unclassified forms of disorders and in 2% (*n* = 1) of healthy individuals.^46^ In our study, NSAbs were more frequent in WD patients than in the control group, but there was no significant difference in NSAbs presence depending on WD manifestations or treatment and the clinical significance of these antibodies in WD remains unclear.

Classical ONA have a greater than 95% association with cancer and their presence together with neurologic symptoms is highly indicative of paraneoplastic neurologic syndromes.^47^ In our study, malignancies were noted in the medical records of 5 WD patients (endometrial cancer, Hodgkin's lymphoma, chronic lymphocytic leukemia, hepatocellular carcinoma and papillary thyroid cancer), but, except in one case, it was not related to the presence of ONA.

In the small number of treatment‐naïve patients, there were no differences in the antibodies studied between the time of diagnosis and 2 years after the treatment initiation. These findings suggest no impact of treatment on autoimmunity in WD, but the results might be influenced by the limited number of patients.

Differences in the frequency of autoantibodies according to the type of *ATP7B* mutation remains unclear and needs further investigation. There is evidence that the H1069Q mutation is associated with a milder disruption of copper metabolism and later WD manifestation comparing to non‐H1069Q mutations.^48^ The limitations of the research are related to the fact that the analysis was made on a small sample of non‐H1069Q patients as it reflects frequency of particular *ATP7B* mutations in Poland. The missense mutation H1069Q is the most common in central, eastern and northern Europe, about 50–80% of patients carry at least one allele with the H1069Q mutation.^2^, ^49^ Hence, it provides the directions for future research that could also investigate the presence of autoantibodies in a larger group of non‐H1069Q patients who are more prevalent in other parts of the world.

The methodology of antibody testing is another potential study limitation. To achieve the highest sensitivity and specificity, it is a recommended practice that results are confirmed with a different laboratory methodology and paired samples of serum and cerebrospinal fluid are tested. The presence of autoantibodies not only in serum, but also in cerebrospinal fluid would increase the specificity and suggest CNS autoimmunity.^50^

## 5 CONCLUSION

In our analysis, we found that the presence of autoantibodies in patients with WD was elevated in comparison to healthy individuals. The role of the autoantibodies in the pathogenesis of WD is unclear, but may possibly relate to a bystander phenomenon. Although there was no apparent link with overt autoimmune diseases in the study, patients with WD, particularly those on D‐penicillamine, should be carefully monitored for autoimmune conditions given the elevated presence of autoantibodies.

### AUTHOR CONTRIBUTIONS

Magdalena Antczak‐Kowalska: laboratory analysis and interpretation of the data, statistical analysis and visualization of the data, writing, revising and approval of the manuscript. Anna Członkowska: planning and supervising the work; writing, revising and approval of the manuscript. Ceren Eyileten: statistical analysis and visualization of the data, writing and approval of the manuscript. Anna Palejko: laboratory analysis and interpretation of the data. Agnieszka Cudna: laboratory analysis and interpretation of the data. Marta Wolska: writing and approval of the manuscript. Agnieszka Piechal: writing and approval of the manuscript. Tomasz Litwin: writing and approval of the manuscript.

The corresponding author attests that all listed authors meet authorship criteria and that no others meeting the criteria have been omitted. All authors have given final approval of the submitted manuscript.

## FUNDING INFORMATION

The work was supported financially as part of the funding for statutory research in the Second Department of Neurology at the Institute of Psychiatry and Neurology, Warsaw, Poland, and the funding for statutory research in the Department of Experimental and Clinical Pharmacology, Medical University of Warsaw, Centre for Preclinical Research and Technology CEPT, Warsaw, Poland.

The authors confirm independence from the sponsors, the content of the article has not been influenced by the sponsors.

## CONFLICT OF INTEREST

The authors declare that they have no conflict of interest.

## ETHICS STATEMENT

The study has been approved by the Ethics Committee of the Institute of Psychiatry and Neurology (4/2018).

## PATIENT CONSENT

All procedures followed were in accordance with the ethical standards of the responsible committee on human experimentation (institutional and national) and with the Helsinki Declaration of 1975, as revised in 2000 (5). Informed consent was obtained from all patients for being included in the study.

## ANIMAL RIGHTS

This article does not contain any studies with animal subjects performed by the any of the authors.

## Data Availability

Additional clinical data not disclosed in this publication will be provided on reasonable request to the corresponding author.
